# Supplementary material for: Molecular Beam Epitaxy of Layered Group III Metal Chalcogenides on GaAs(001) Substrates
Source: Materials (Basel). 2020 Aug 5;13(16):3447. doi: 10.3390/ma13163447 (PMC7475857; doi:10.3390/ma13163447)
Supplement: Supplementary file 1 [file materials-13-03447-s001.pdf]

materials

Supplementary

Molecular Beam Epitaxy of Layered Group III Metal Chalcogenides on GaAs(001) Substrates

Sergey V. Sorokin[1,*], Pavel S. Avdienko[1], Irina V. Sedova[1], Demid A. Kirilenko[1], Valery Yu. Davydov[1], Oleg S. Komkov[2], Dmitrii D. Firsov[2] and Sergey V. Ivanov[1,*]

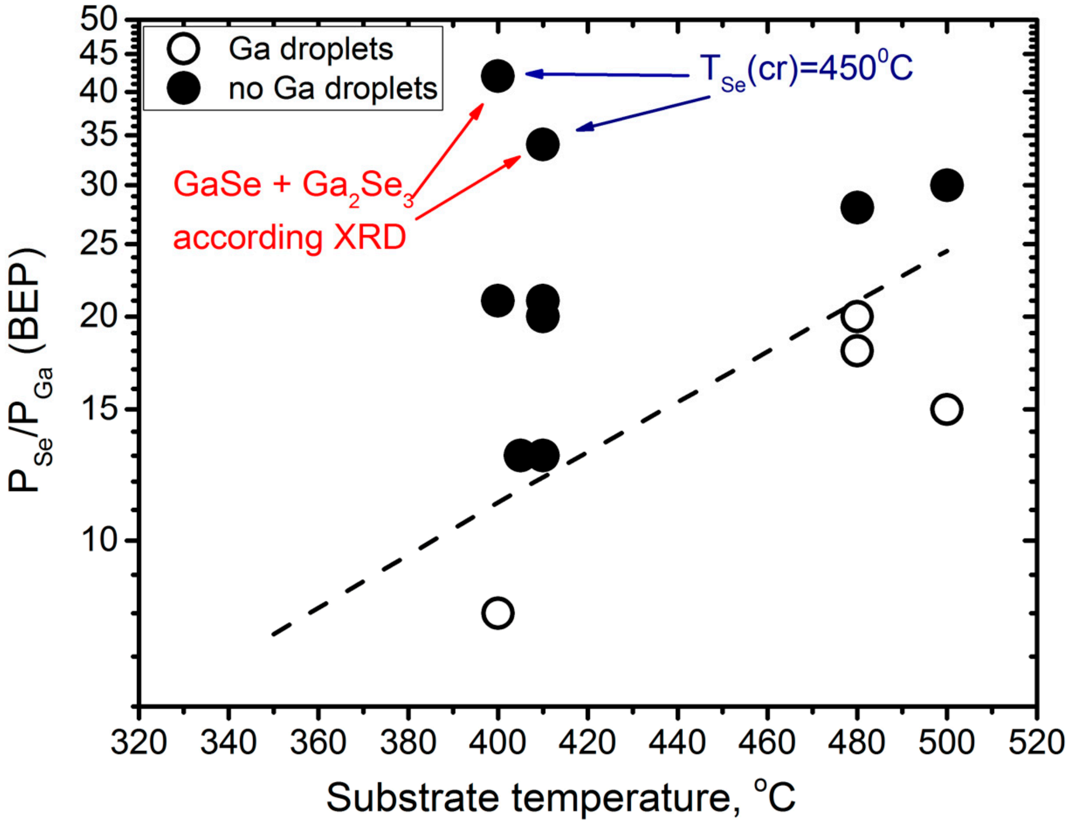

Figure S1. The dependence of surface morphology of GaSe/GaAs(001) layers grown using standard Ga and Se valve cracking cell with T_{Se}(cr) = 500 °C on both P_{Se}/P_{Ga} (BEP) ratio and substrate temperature. GaSe layers with and without Ga droplets on the growth surface are indicated by the hollow and filled circles.

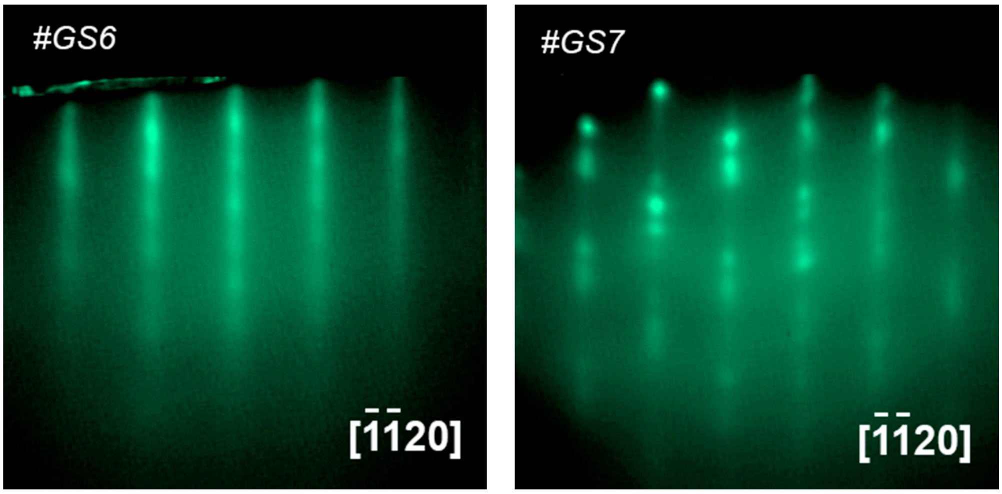

(a) (b)

Figure S2. RHEED patterns during the growth of GaSe layers: (**a**) *#GS6*, T_S ~ 400 °C, Se/Ga (BEP) = 12, r_{GaSe} ~ 1.5 nm/min; and (**b**) *#GS7*, T_S ~ 500 °C, Se/Ga (BEP) = 25, r_{GaSe} ~ 2.1 nm/min.

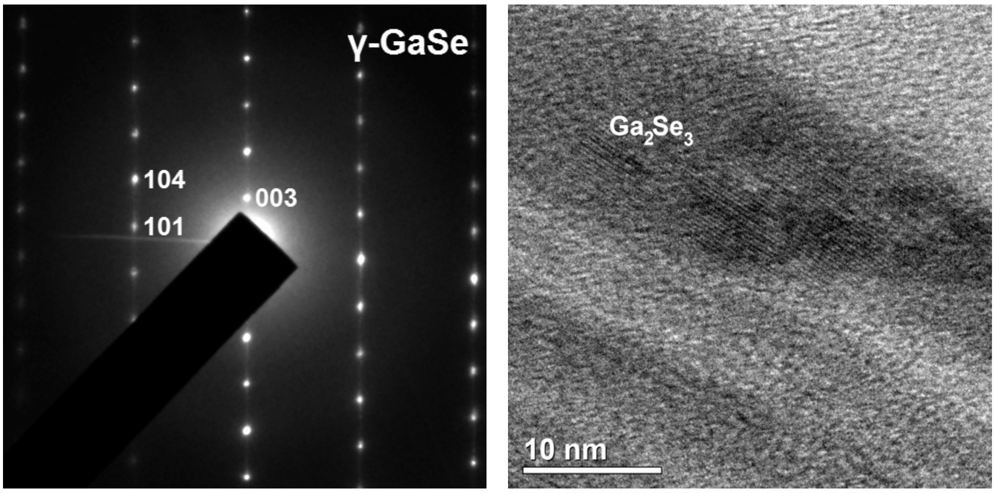

(a) (b)

Figure S3. (**a**) The selective area electron diffraction pattern of GaSe layer grown on a GaAs(001) substrate at T_S ~ 400 °C under strong Se-rich conditions (#GS1); and (**b**) cross-section TEM image, demonstrating an existence of the Ga_2Se_3 phase inclusions in the same layer.

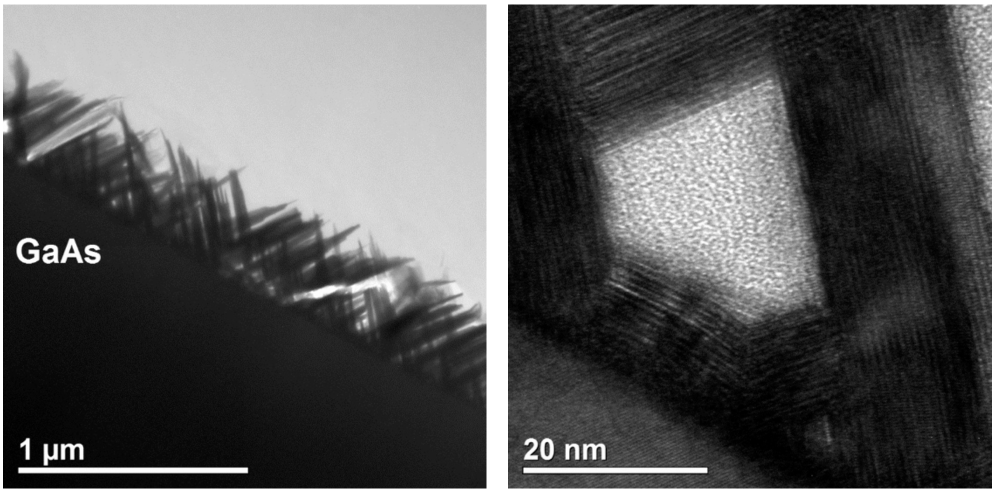

(a) **(b)**

Figure S4. Cross-section TEM (**a**) and HRTEM (**b**) images of the GaSe layer grown on GaAs(001) substrate at T_S ~ 500 °C (Se/Ga(BEP) ~ 25) (sample #*GS7*).

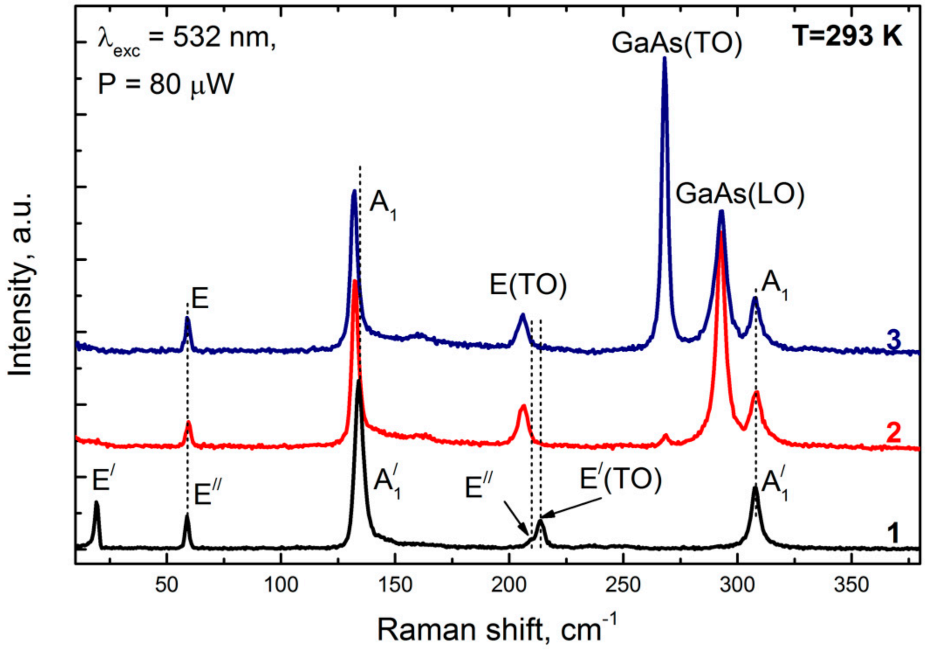

Figure S5. Raman spectrum of the bulk ε-GaSe layer produced by HQ Graphene (Curve 1). Typical Raman spectra of γ-GaSe layers grown on GaAs(001) and GaAs(112) substrates at T_S ~ 400 °C (Curves 2 and 3, respectively). All spectra are normalized to the intensity of the A_1 (132 cm^{-1}) phonon line.

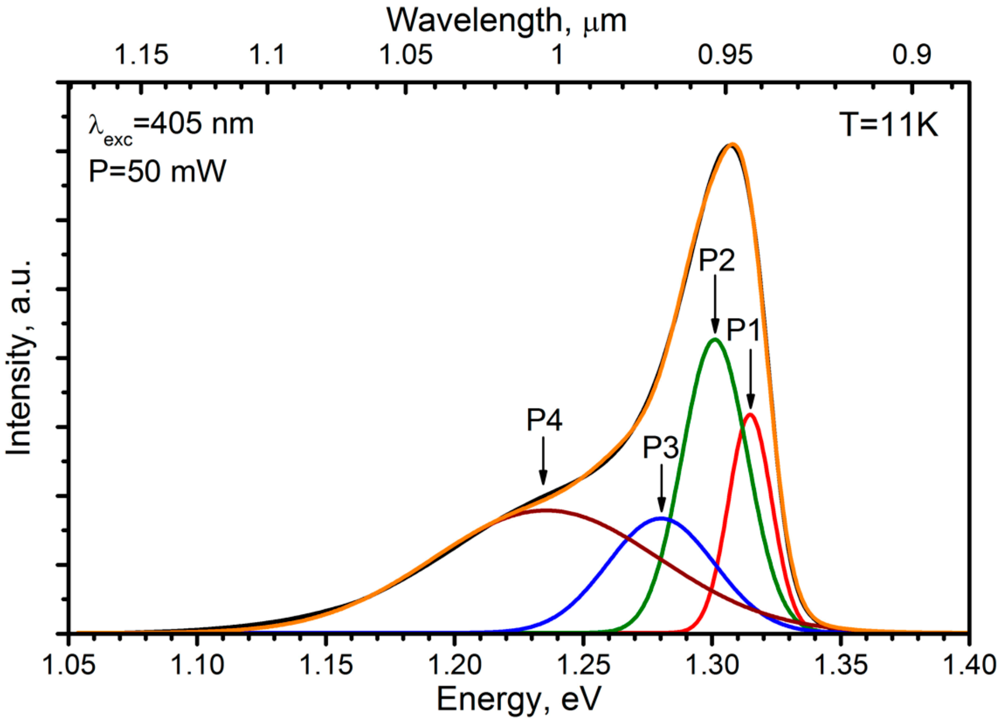

Figure S6. Photoluminescence spectrum of the InSe/GaAs(001) layer measured at T = 11 K. The spectrum is most accurately approximated by four Gaussian contours centered at 1.315, 1.301, 1.28, and 1.2 eV, respectively.

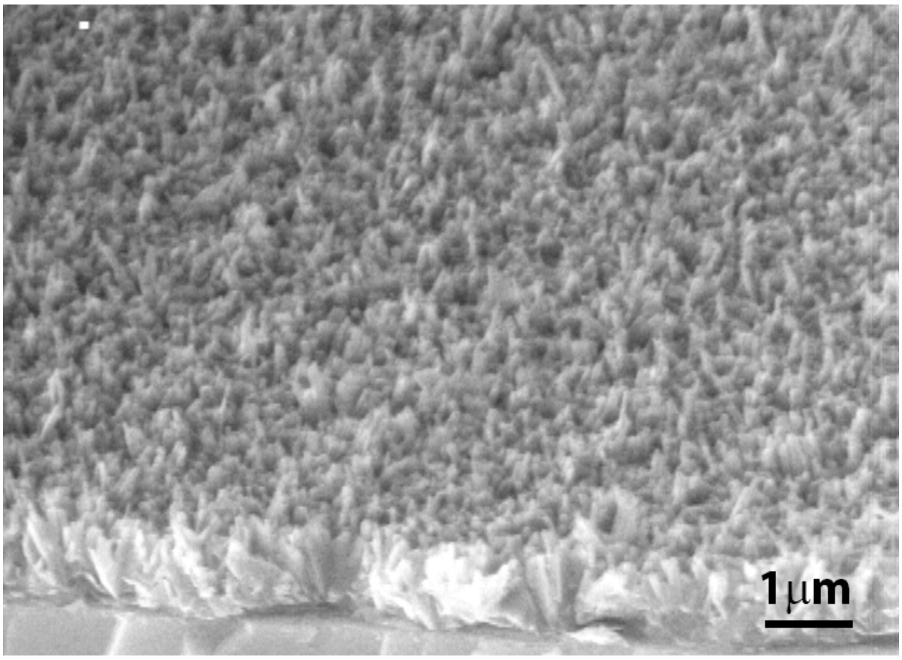

Figure S7. The plan-view SEM image of the InSe/GaSe/GaAs(001) structure. The growth temperature of InSe layer was as high as T_S ~ 450 °C.

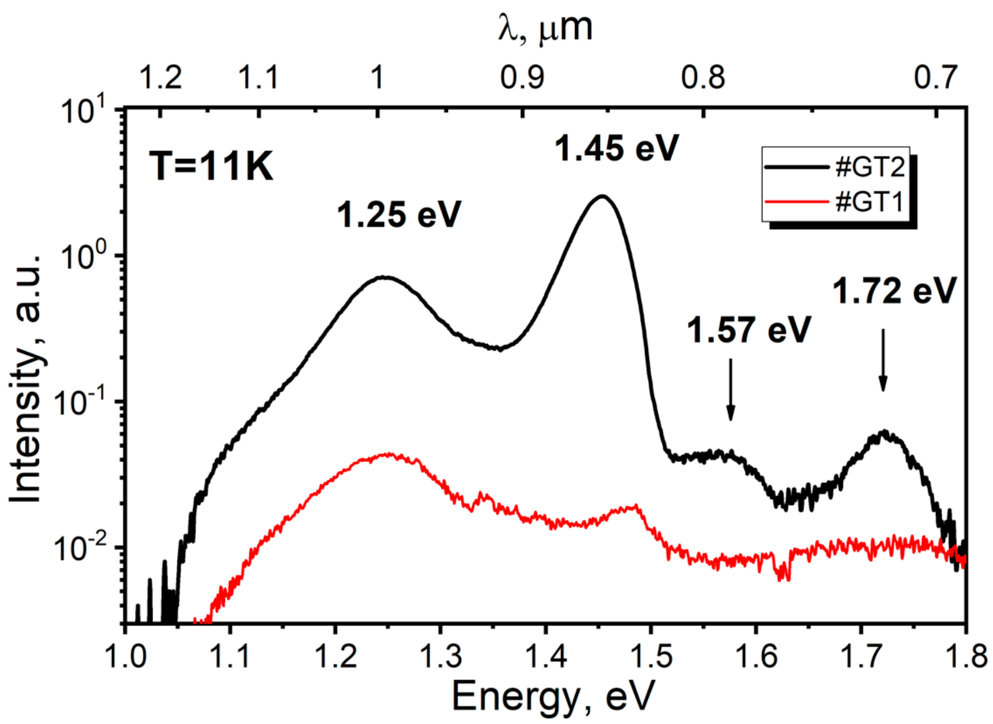

Figure S8. PL spectra of the GaTe/GaAs(001) layers #*GT1* and #*GT2* measured at the same excitation power density of 0.5 W/cm^2.

© 2020 by the authors. Submitted for possible open access publication under the terms and conditions of the Creative Commons Attribution (CC BY) license (http://creativecommons.org/licenses/by/4.0/).
